# High risk of burnout syndrome and associated factors in medical students: A cross-sectional analytical study

**DOI:** 10.1371/journal.pone.0304515

**Published:** 2024-05-31

**Authors:** Irena Ilic, Ivana Zivanovic Macuzic, Milena Ilic

**Affiliations:** 1 Faculty of Medicine, University of Belgrade, Belgrade, Serbia; 2 Department of Anatomy, Faculty of Medical Sciences, University of Kragujevac, Kragujevac, Serbia; 3 Department of Epidemiology, Faculty of Medical Sciences, University of Kragujevac, Kragujevac, Serbia; United Arab Emirates University, UNITED ARAB EMIRATES

## Abstract

**Introduction:**

Although research on burnout syndrome in medical students has increased in recent years, results are inconsistent about which factors are associated with a high risk for burnout syndrome. The aim of this study was to assess the prevalence of high risk of burnout syndrome and to identify factors associated with burnout in medical students in preclinical and clinical training.

**Method:**

A cross-sectional study was conducted at the University of Kragujevac, Serbia. The Maslach Burnout Inventory Student Survey and an epidemiological questionnaire on basic socio-demographic and academic characteristics were used. Statistical evaluation was performed through logistic regression analysis, using Odds Ratio (OR) and 95% Confidence Interval (CI).

**Results:**

Among medical students, no statistically significant differences were found in the prevalence of high risk of burnout syndrome in preclinical (14.8%) and clinical grade (15.1%), p > 0.05. High risk for burnout syndrome in preclinical study years was independently associated with the female sex (adjusted OR = 0.41, 95%CI = 0.19–0.91, p = 0.028), and cigarette smoking (adjusted OR = 2.47, 95%CI = 1.05–5.78, p = 0.038). The high risk of burnout syndrome was associated with sedatives use (adjusted OR = 4.03, 95%CI = 1.27–12.73; p = 0.018) only in clinical years medical students. The frequency of alcohol consumption was correlated with the high risk of burnout syndrome in medical students in both preclinical and clinical training, but without statistical significance (both p for trend < 0.1).

**Conclusion:**

There was a significant prevalence of burnout among medical students, with some modifiable associated factors revealed.

## Introduction

Emotional exhaustion, depersonalization, and decreased feeling of personal accomplishment are the triad that makes up burnout syndrome [1]. It can happen in persons working with people [1]. Even though it was considered a syndrome characteristic for “helping” professionals [2, 3], the syndrome has recently been expanded to involve all occupational groups, including students [4]. Burnout syndrome is frequent among students, but it has been suggested that medical students are more prone to burnout than others [5]. According to systematic reviews of the literature, approximately 50% of medical students experience burnout [6, 7]. Compared to other students, medical students have experienced emotional exhaustion, depersonalization and overall burnout significantly more often [8].

Over the past years, the research concerning burnout in medical students has been increasing, although most of it focused on the clinical level of studies and only several have focused on the preclinical level of studies. The research in the medical students population showed that the prevalence of burnout is like ladders, with students of preclinical study years taking the first step with the lowest burnout prevalence. The total burnout syndrome prevalence ranged between 2% and 76%, based on the definition and used criteria, investigated samples and used cut-off: students in preclinical years 2–53% [9–12], and students in clinical years 10–45% [13–15]. Extreme burden and academic curriculum, significant educational demands, paucity of time to rest and for friends and family, exams, and specialty selection, combined with financial demands and delayed income, contribute to the stress in medical students [16, 17]. Transition from the introductory, preclinical studies into the final, clinical studies can represent another opportunity for intensive uncertainty, anxiety, expectations and fears due to medical student’s sense of being limited with scientific knowledge, but also due to the immediate contact with severely ill persons with despairing prognosis. Emotional exhaustion due to facing disease and death is a professional stressor that medical students encounter during intensive clinical practice [18]. Contrary to some authors [19], Alqahtani and coauthors [20] did not record a significant association of burnout syndrome prevalence with study year.

Significant consequences at the individual level, such as quitting the studies, making bad decisions, unprofessional conduct, hostility towards the patients, medical errors, bad relations with colleagues, depression, anxiety, tiredness, sleeping disorders, alcoholism, drug abuse and suicidal ideas point towards the importance of researching burnout in medical students [10, 21, 22]. Besides, the increased level of burnout was significantly associated with poor quality of life, hypertension and other disorders of the cardiovascular system.

Since the 1980s, the majority of studies about burnout have been carried out in developed countries and there is still insufficient knowledge of the issue of burnout among medical students in some countries with limited resources. The gap in the literature on burnout among medical students remains a big challenge because students in the twenty-first century are facing increasing demands to acquire as much knowledge and skills as quickly as possible, as confirmed by the recent COVID-19 pandemic. In addition to the fact that the results of previous studies indicate large differences in the frequency of burnout among medical students that have not yet been satisfactorily evaluated, the findings on risk factors for burnout are not consistent either. Consequently, to date, there is no consensus on recommendations for mitigating burnout in medical students using evidence-based medicine. Hence, research on the frequency of burnout among medical students and the factors associated with it can give the necessary attention to this current problem and increase the body of knowledge that will enable finding approaches for the successful prevention of burnout syndrome.

There is not enough evidence regarding burnout in Serbian medical students [23, 24], whereby only one of those studies applied a validated tool that was recommended for research in the student population, while the other study also explored the mental health status of medical students. During the past decades, medical sciences have been developing rapidly, which, considering that burnout has not been researched enough in medical students in our environment, points out the necessity of estimating the prevalence of burnout in medical students and exploring possible associations of burnout and socio-demographic and academic characteristics of medical students. The aim of the study was to assess the prevalence of high risk for burnout and to identify factors associated with burnout in medical students of preclinical and clinical training.

## Methods

### Setting

The Faculty of Medical Sciences in Kragujevac is a state medical faculty, founded in 1977. The faculty organizes studies at the level of integrated academic studies through 12 blocks (six study years), and subjects are taught via lectures, exercises, other forms of teaching and professional practice. Since 2010, this faculty began enrolling about 90 medical students per year to meet the curriculum quality standards, so the number of students was smaller only up to the third academic year. To obtain the medical doctor title, the students must pass 35 mandatory, 6 out of 15 offered elective courses, complete professional practice and defend the final exam.

### Study design

This study was cross-sectional. The collected data were self-reported. All students of medicine were handed the questionnaires during the classes, alongside a cover letter that contained all information about the research and a consent form.

### Study population

All medical students enrolled in the study year as regular students were invited to participate in the study. The study population is represented by medical students who enrolled at the university as follows: one group enrolled in studies while non-complete Bologna was applied (later years) and another group enrolled in studies when complete Bologna was applied (earlier years).

### Study sample and sample size calculation

Students included in the study were medical students of all six years of studies at the Faculty of Medical Sciences in Kragujevac, who accepted voluntarily to take part in the research by signing the informed consent form. The criteria for inclusion were: age > 18 years, regular student status, lecture attendance, signed written informed consent to participate and lack of criteria for exclusion. The criteria for exclusion were: age < 18 years and absence from regular lectures.

We used the Epi Info StatCalc software (Centers for Disease Control and Prevention, Atlanta, Georgia, USA) to calculate the sample size. By entering the following parameters: population size -836, 5%—margin of error, expected frequency—22.6% [25] and confidence level 99.99%, the minimum sample size was determined as 467. After adjusting for a potential non-response rate (15%), it was calculated that the minimum sample size was 538. In total, 760 participants out of 836 students voluntarily completed the questionnaire (participation rate = 90.9%).

### Ethical considerations

This study was part of research that was approved by the Ethics Committee of the Faculty of Medical Sciences, University of Kragujevac (Ref. No.: 01–1176). Informed, written, voluntary consent was provided by all study participants before taking part in the study.

### Data collection

Along with the epidemiological questionnaire (questionnaire about socio-demographic characteristics, academic performance, habits, personal medical history, etc.), this research also used the Maslach Burnout Inventory—Student Survey to assess the level of risk for burnout syndrome in medical students [4]. The questionnaire was filled out by students (paper and pencil), and filling out was done in amphitheater and lecture auditoriums at the Faculty of Medical Sciences in Kragujevac. Medical doctors/authors of this manuscript were present while the students answered the questionnaires. Medical students had about 15(± 5) minutes to fill out the study survey. This research was conducted from 15 February to 14 March 2014.

### Instruments

The epidemiologic questionnaire contained questions regarding socio-demographic characteristics, i.e. sex (male / female), completed secondary school (grammar school / medical and other schools), marital status (with partner / without partner), whether they have children, achieved academic results (cumulative total average grade ≤ 8 / > 8; repeated year (No / Yes), level of studies (medical students of the first-third year: preclinical study level; students of the fourth-sixth year: clinical study level, study financing (state-sponsored / self-funded), housing (in own home/ with parents / as subtenants / in student dormitory), habits (smoking cigarettes, drinking alcohol) and personal medical history (presence of any chronic disease), use of sedatives. If a student regularly smoked at least a cigarette a day during one year they were considered smokers; participants who smoked at least one cigarette every day during the last 12 months were considered current smokers, and those for whom it has been at least a year since they stopped smoking as former smokers.

The Maslach Burnout Inventory–Student Survey (MBI-SS), as an instrument that assesses the level of burnout among students, was constructed by [4]. MBI-SS has 15 items that measure the three dimensions of burnout: Emotional Exhaustion–EX (5 items), Cynicism–CY (4 items), and Academic Efficacy–AE (6 items). The questions refer to how one feels about university and their academic work. The level of agreement with each item is scored on a 7-point Likert-type scale that ranges from 0 to 6 (0—never, 1 –several times a year, 2 –once a month, 3 –several times a month, 4 –once a week, 5 –several times a week, 6 –every day), to describe best how often one feels in a certain way.

Our study confirmed the validity and reliability of the original MBI-SS scale and its 3-factor structure, with the Cronbach’s α coefficients for Emotional Exhaustion, Cynicism, and Academic Efficacy of 0.869, 0.856, and 0.852, respectively [26]. High level of burnout was identified as: emotional exhaustion over 14, cynicism over 6 and professional efficacy less than 23 [4, 5, 27, 28].

### Statistical analysis

We used descriptive and analytical statistics to analyze data. Questionnaires that were not completely filled in or were inadequately filled in were not included in this analysis. All categorical variables were presented as proportions (percentages). The difference between preclinical and clinical students of medicine according to the prevalence of burnout syndrome was examined using the chi-square test. We used univariate and multivariate logistic regression analyses to determine Odds Ratio (OR) with 95% Confidence Interval (95%CI) to examine the association between characteristics of medical students and high risk for burnout. Next, we adjusted ORs for all variables related to burnout in the univariate analyses at p < 0.10. Also, adjustment was made for variables that were associated with burnout (residence, marital status, children, housing, completed secondary school, study financing, cumulative total average grade, re-enrollment in the academic year, length of study, sports, recreational activity) according to literature data, even though they did not differ significantly between preclinical and clinical medical students in this study. We did not include age in the multivariate models due to its collinearity with some of the study years. The Hosmer-Lemeshow test of goodness of fit and Cox and Snell’s and Nagelkerke’s Pseudo R square measures were used to estimate model fit. The linear trend in the risk test was based on the logistic regression model. All statistical analyses were performed separately for medical students in preclinical and clinical study years. We used the SPSS Software (SPSS Inc, version 20, Chicago, IL) to perform all statistical analyses.

## Results

### Research flow diagram

**Fig 1:**
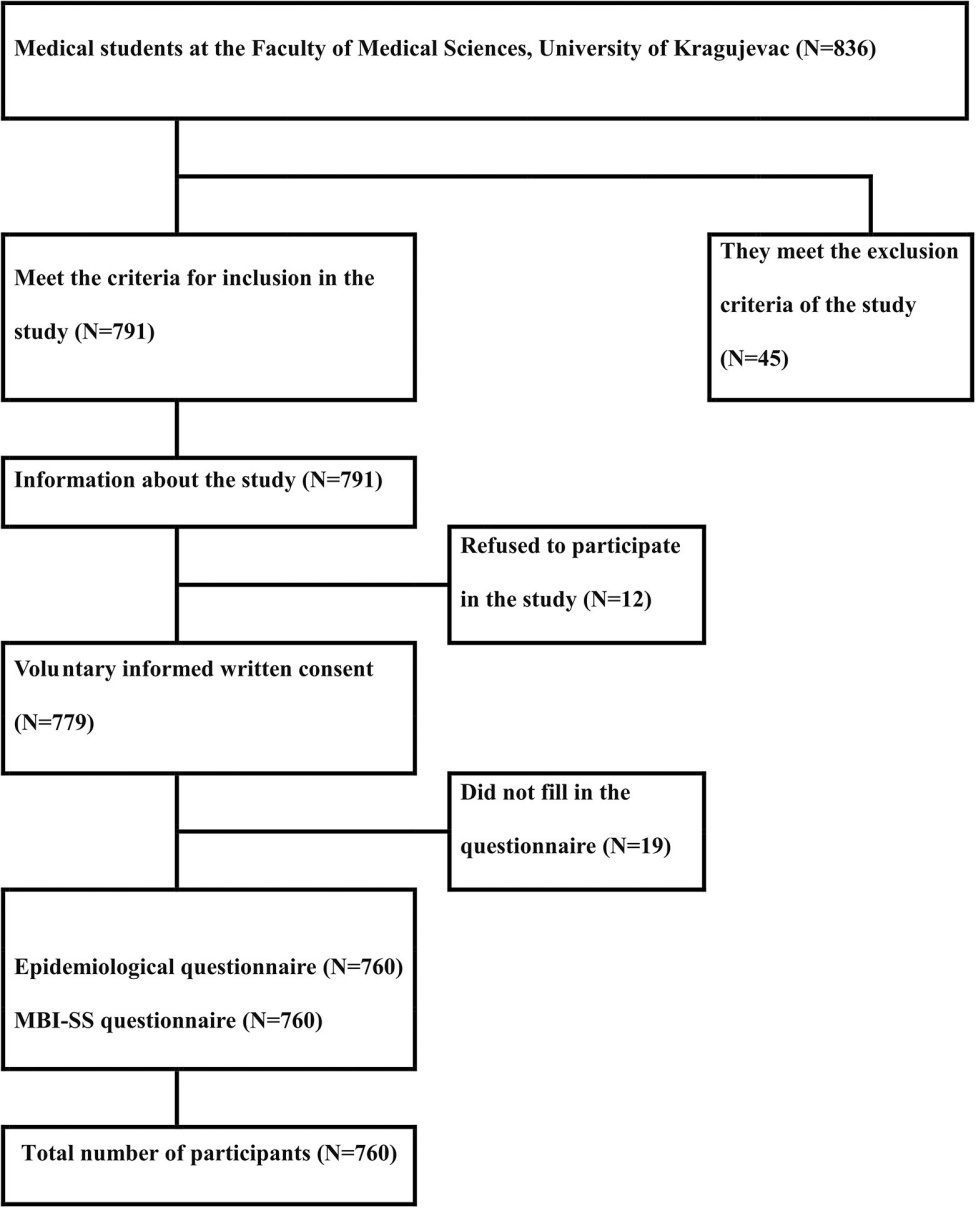
Research flow diagram.

The study included 760 out of 836 medical students who met the participation criteria. Absence from regular classes was the main reason for exclusion from the study (45 students) (Fig 1). After being informed about the research, out of the total number of medical students who met the criteria for inclusion in the study (791 students), 12 students refused to participate in the study. The reason for not accepting or refusing to participate in the study was most often a lack of interest in the study. After signing the voluntary informed consent to participate in the study (779 students), some subjects did not return the questionnaire or did not complete the questionnaire during recruitment for the study, or the questionnaires were not fully completed (19 students in total).

### Basic characteristics of preclinical and clinical group of medical students

A total of 64.6 percent of medical students were females, equally in preclinical and clinical study years (Table 1). Among the students in clinical study years, there were more of those who finished medical secondary school (68.5%) than in students in preclinical studies (60.6%). Only 2 (0.7%) medical students in preclinical studies had children at the time of the survey, and 20 (4.2%) students in clinical studies. Clinical medical students were more often living as subtenants, compared to preclinical students (48.7% vs. 40.1%). More preclinical (14.4%) than clinical students (7.6%) lived in student dormitories. Preclinical students had their studies more often state-sponsored (95.1%) than clinical students (68.1%). Compared to preclinical students, those in clinical studies more often repeated year at studies (37.6% vs. 2.1%). Most (86.3%) of the preclinical medical students had a cumulative average grade higher than 8, compared to 50.2% of clinical studies students. There were more smokers among the students of clinical than preclinical study years (36.8% vs. 23.9%), and that were mostly former smokers (16.2% vs. 8.1%). About 60% of medical students both in preclinical and clinical study years consumed alcohol. The students did not differ according to positive personal medical history for chronic diseases and sedatives use.

**Table 1:** Basic characteristics of preclinical and clinical group of medical students.

		Preclinical (N = 284)	Clinical (N = 476)
Variables		Number (%)	Number (%)
**Sex**			
	Male	105 (37.0)	164 (34.5)
	Female	179 (63.0)	312 (65.5)
**Completed secondary school**			
	Grammar school	112 (39.4)	150 (31.5)
	Medical school	172 (60.6)	326 (68.5)
**Marital status**			
	With partner	115 (40.5)	207 (43.5)
	Without partner	169 (59.5)	269 (56.5)
**Child**			
	No	282 (99.3)	456 (95.8)
	Yes	2 (0.7)	20 (4.2)
**Study financing**			
	State-sponsored	270 (95.1)	324 (68.1)
	Self-funded	14 (4.9)	152 (31.9)
**Housing**			
	In own home	26 (9.2)	44 (9.2)
	With parents	103 (36.3)	164 (34.5)
	As subtenants	114 (40.1)	232 (48.7)
	In student dormitory	41 (14.4)	36 (7.6)
**Cumulative total average grade**			
	Low	39 (13.7)	237 (49.8)
	High	245 (86.3)	239 (50.2)
**Repeat-year students**			
	No	278 (97.9)	297 (62.4)
	Yes	6 (2.1)	179 (37.6)
**Cigarette smoking**			
	Never	216 (76.1)	301 (63.2)
	Ever	68 (23.9)	175 (36.8)
**Smoking status**			
	Non smokers	216 (76.1)	301 (63.2)
	Former smokers	23 (8.1)	77 (16.2)
	Current smokers	45 (15.8)	98 (20.6)
**Alcohol consumption**			
	No	106 (37.3)	199 (41.8)
	Yes	178 (62.7)	277 (58.2)
**Frequency of alcohol consumption**			
	Non drinkers	106 (37.3)	199 (41.8)
	1–2 times a year	28 (9.9)	38 (8.0)
	1–2 times a month	113 (39.8)	189 (39.7)
	1–2 times a week	32 (11.3)	45 (9.5)
	Every day	5 (1.8)	5 (1.1)
**Positive personal medical history**			
	No	264 (93.0)	450 (94.5)
	Yes	20 (7.0)	26 (5.5)
**Use of sedatives**			
	No	274 (96.5)	460 (96.6)
	Yes	10 (3.5)	16 (3.4)

### High risk of burnout syndrome in medical students

According to the MBI-SS subscales, a high risk of emotional exhaustion is recorded in 308/760 medical students (in preclinical: 129–41.9%; in clinical: 179–58.1%; p = 0.034) (Table 2). The cynicism subscale showed a high risk of burnout in 266/760 (i.e. 35.0%) participants (in preclinical: 75–28.2%; in clinical: 191–71.8%; p = 0.000). The academic inefficacy subscale showed that 287/760 (i.e. 37.7%) of students were in the category of high risk of burnout (in preclinical: 81–28.2%; in clinical: 206–71.8%; p = 0.000).

**Table 2:** High risk of burnout syndrome in preclinical and clinical group of medical students (N = 760).

		Burnout syndrome–high risk			
		Preclinical	Clinical		
MBI-SS subscales		N (%)	N (%)	Total	P
**MBI EE**		129 (41.9)	179 (58.1)	308 (40.5)	0.034
**MBI CY**		75 (28.2)	191 (71.8)	266 (35.0)	0.000
**MBI rAE**		81 (28.2)	206 (71.8)	287 (37.7)	0.000
**High risk for burnout syndrome–Total**		42 (14.8)	72 (15.1)	114 (15.0)	0.900

MBI-SS (Maslach Burnout Inventory—Student Survey); MBI EE (Emotional Exhaustion); MBI CY (Cynicism); MBI rAE (reverese Аcademic Еfficacy).

p (probability, χ^2^-test).

Among medical students, there were no statistically significant differences in the prevalence of high risk for burnout in preclinical (14.8%) and clinical grade (15.1%), p = 0.900.

### Characteristics of preclinical and clinical group of medical students with high risk of burnout

In preclinical study years, the univariate logistic regression pointed to the potential significance that female sex and alcohol consumption of 1–2 times a month could have on the high risk for burnout (both p < 0.1) (Table 3). Based on the results of univariate logistic regression (model for clinical study years), the re-enrollment of the academic year, cigarette smoking and positive medical history (all equally p < 0.1) could potentially be significant for high risk of burnout in medical students, while for the alcohol consumption of 1–2 times a week OR was 3.36 (95%CI = 1.27–8.94; p = 0.015) and for use of sedatives OR was 5.51 (95%CI = 2.13–14.30; p < 0.000).

**Table 3:** Characteristics of preclinical and clinical group of medical students with high risk of burnout syndrome.

		Preclinical (N = 284)						Clinical (N = 476)					
		Burnout syndrome (high risk)						Burnout syndrome (high risk)					
		Absent	Present					Absent	Present				
Variables		N (%)	N (%)		OR (95% CI)	p*		N (%)	N (%)		(95% CI)	p*	
**Sex**													
	Male	84 (34.7)	21 (50.0)		1.00**			134 (33.2)	30 (41.7)		1.00**		
	Female	158 (65.3)	21 (50.0)		0.53 (0.28–1.03)	0.061		270 (66.8)	42 (58.3)		0.70 (0.41–1.16)	0.164	
**Completed secondary school**													
	Grammar school	96 (39.7)	16 (38.1)		1.00**			127 (31.4)	23 (31.9)		1.00**		
	Medical school	146 (60.3)	26 (61.9)		1.07 (0.55–2.10)	0.847		277 (68.6)	49 (68.1)		0.98 (0.57–1.67)	0.932	
**Marital status**													
	With partner	76 (34.9)	18 (35.3)		1.00**			198 (46.3)	30 (47.6)		1.00**		
	Without partner	142 (65.1)	26 (61.9)		0.98 (0.52–1.86)	0.954		230 (53.7)	33 (52.4)		0.95 (0.56–1.61)	0.840	
**Child**													
	No	240 (99.2)	42 (100.0)		1.00**			387 (95.8)	69 (95.8)		1.00**		
	Yes	2 (0.8)	0 (0.0)		--	--		17 (4.2)	3 (4.2)		0.99 (0.28–3.47)	0.987	
**Study financing**													
	State-sponsored	232 (95.9)	38 (90.5)		1.00**			279 (69.1)	45 (62.5)		1.00**		
	Self-funded	10 (4.1)	4 (9.5)		2.44 (0.73–8.18)	0.148		125 (30.9)	27 (37.5)		1.34 (0.80–2.26)	0.272	
**Housing**													
	In own home	21 (8.7)	5 (11.9)		1.00**			40 (9.9)	4 (5.6)		1.00**		
	With parents	86 (35.5)	17 (40.5)		0.83 (0.28–2.51)	0.741		143 (35.4)	21 (29.2)		1.47 (0.48–4.53)	0.503	
	As subtenants	101 (41.8)	3 (31.0)		0.54 (0.17–1.68)	0.288		187 (46.3)	45 (62.5)		2.41 (0.82–7.07)	0.110	
	In student dormitory	34 (14.0)	7 (16.6)		0.87 (0.24–3.08)	0.822		34 (8.4)	2 (2.8)		0.59 (0.10–3.41)	0.554	
**Cumulative total average grade**													
	Low	76 (34.9)	24 (47.1)		1.00**			150 (35.0)	26 (41.3)		1.00**		
	High	142 (65.1)	27 (52.9)		0.60 (0.33–1.12)	0.109		278 (65.0)	37 (58.7)		0.77 (0.45–1.32)	0.337	
**Repeat-year students**													
	No	163 (74.8)	33 (64.7)		1.00**			336 (78.5)	43 (68.3)		1.00**		
	Yes	55 (25.2)	18 (35.3)		1.62 (0.84–3.10)	0.148		92 (21.5)	20 (31.7)		1.70 (0.95–3.03)	0.073	
**Cigarette smoking**													
	Never	138 (63.3)	31 (60.8)		1.00**			309 (72.2)	39 (61.9)		1.00**		
	Ever	80 (36.7)	20 (39.2)		1.11 (0.60–2.08)	0.738		119 (27.8)	24 (38.1)		1.60 (0.92–2.77)	0.095	
**Smoking status**													
	Non smokers	138 (63.3)	31 (60.8)		1.00**			309 (72.2)	39 (61.9)		1.00**		
	Former smokers	40 (18.3)	8 (15.7)		0.89 (0.38–2.09)	0.790		42 (9.8)	10 (15.9)		1.89 (0.88–4.06)	0.104	
	Current smokers	40 (18.3)	12 (23.5)		1.34 (0.63–2.84)	0.452		77 (18.0)	14 (22.2)		1.44 (0.75–2.79)	0.278	
	*** p for trend					0.679						0.202	
**Alcohol consumption**													
	No	52 (23.9)	15 (29.4)		1.00**			209 (48.8)	29 (46.0)		1.00**		
	Yes	166 (76.1)	36 (70.6)		0.75 (0.38–1.48)	0.410		219 (51.2)	34 (54.0)		1.12 (0.66–1.90)	0.678	
**Frequency of alcohol consumption**													
	Non drinkers	52 (23.9)	15 (29.4)		1.00**			209 (48.8)	29 (46.0)		1.00**		
	1–2 times a year	13 (6.0)	2 (3.9)		0.53 (0.11–2.63)	0.440		48 (11.2)	3 (4.8)		0.45 (0.13–1.54)	0.204	
	1–2 times a month	109 (50.0)	16 (31.4)		0.51 (0.23–1.11)	0.089		154 (36.0)	23 (36.5)		1.08 (0.60–1.93)	0.805	
	1–2 times a week	40 (18.3)	15 (29.4)		1.30 (0.57–2.97)	0.534		15 (3.5)	7 (11.1)		3.36 (1.27–8.94)	0.015	
	Every day	4 (1.8)	3 (5.9)	2.60 (0.52–12.92)			0.243	2 (0.5)	1 (1.6)		3.60 (0.32–41.00)	0.301	
	*** p for trend					0.076						0.052	
**Positive personal medical history**													
	No	210 (96.3)	49 (96.1)		1.00**			400 (93.5)	55 (87.3)		1.00**		
	Yes	8 (3.7)	2 (3.9)		1.07 (0.22–5.20)	0.932		28 (6.5)	8 (12.7)		2.08 (0.90–4.79)	0.086	
**Use of sedatives**													
	No	212 (97.2)	50 (98.0)		1.00**			417 (97.4)	55 (87.3)		1.00**		
	Yes	6 (2.8)	1 (2.0)		0.71 (0.08–6.00)	0.750		11 (2.6)	8 (12.7)		5.51 (2.13–14.30)	0.000	

* p–probability, value according to univariate logistic regression analysis

** Reference category

*** p for trend (according to logistic regression).

Abbreviations: OR–Odds Ratio; 95% CI—95% Confidence Interval.

### Associated factors of high risk of burnout in preclinical and clinical medical students

Significant independent predictors of high risk of burnout in medical students in preclinical study years based on the multivariate logistic regression analysis results were female sex (adjusted OR = 0.41; 95%CI = 0.19–0.91; p = 0.028) and smoking cigarettes (adjusted OR = 2.47; 95%CI = 1.05–5.78; p = 0.038) (Table 4). Multivariate logistic regression analysis showed that the use of sedatives was a significant independent predictor of high risk of burnout in medical students at clinical study years (adjusted OR = 4.03; 95%CI = 1.27–12.73; p = 0.018).

**Table 4:** High risk of burnout in preclinical and clinical group of medical students: Multivariate logistic regression.

Group	Variables		Adjusted* OR	95% CI	p
**Preclinical**					
	**Sex**				
		Male	1**		
		Female	0.41	0.19–0.91	0.028
	**Cigarette smoking**				
		Never	1**		
		Ever	2.47	1.05–5.78	0.038
**Clinical**					
	**Use of sedatives**				
		No	1**		
		Yes	4.03	1.27–12.73	0.018

* Adjusted for residence, marital status, children, housing, completed secondary school, study financing, cumulative total average grade, re-enrollment in the academic year, length of study, cigarette smoking, alcohol consumption, sports, recreational activity, positive personal medical history, use of sedatives

** Reference category.

Abbreviations: OR–Odds Ratio; 95% CI—95% Confidence Interval; p–probability, value according to multivariate logistic regression analysis.

## Discussion

The present study represents one of the first attempts to estimate the risk level for burnout among medical students in Serbian to analyze the role of demographic characteristics and academic performance of students in burnout. This research revealed that medical students had a significant prevalence of high risk of burnout syndrome. Most of the variables associated with medical students’ burnout are potentially preventable.

A high risk of burnout in medical students enrolled at the Faculty of Medical Sciences in Kragujevac was recorded in 15.0% of participants, i.e. in 14.8% of students of preclinical and in 15.1% of students of clinical years. According to the meta-analysis of literature in English and German language published in the 2000–2017 period in countries worldwide, prevalence rates of burnout syndrome in medical students ranged from 7.0% to 75.2% [29]. At one medical faculty in Brazil, research showed that 70.6% of first to fourth-year medical students experienced a high level of emotional exhaustion, nearly half showed cynicism (52.8%) and 48.7% showed academic inefficacy, which led to burnout prevalence of 26.4% [30]. These results are similar to that of other international research according to which, on average, the burnout prevalence ranged from 21% to 71% [12, 13, 31]. Medical students experience a high level of burnout due to the increasing difficulty of the curriculum and program of studies that is placed upon students, followed by frequent contact with people with severe diseases and death, as well as routinely experiencing a discord between the rational and emotional [32, 33]. The observed differences in the prevalence of burnout in medical students could be due to the differences in cultures, socio-economic status and studied population. Further on, some research included only students in the 3^rd^ and 4^th^ year of studies, and the present study incorporated medical students of all six study years. Besides, differences in the observed prevalence of burnout in medical students could be due to the use of different questionnaires for the assessment of burnout and sample size. Comparing the results of this study with literature data can be difficult due to many reasons, including the use of different measuring instruments and different cut-off values for burnout assessment, using different criteria for defining burnout, significant variability in medical studies curriculums between universities, etc.

In some previous studies, the prevalence of burnout increased with advancing medical study years [12, 13], while some studies [7, 20, 34] did not note this. A study in Lebanon [8] recorded that medical students enrolled in the first study year had burnout syndrome significantly more often. Some authors link this difference with graduate and better adjustment of students of higher years of studies of medicine to the new environment leading to decreased burnout risk [35]. In this research, medical students of the first three study years enrolled to faculty according to the Bologna system (this is the first generation of students enrolled according to the new Bologna program from the beginning of their studies), while the students in the fourth, fifth and sixth years studies according to the classical studying system. To some extent, the third-year students are an exemption, among whom the majority are students enrolled according to the Bologna system and a minority of them are students who have renewed the school year while studying previous years according to the classical system of schooling. A possible explanation for the absence of a significant association obetween the high risk for burnout and studies in preclinical/clinical subjects should be looked for in that context in this and similar research [36], contrary to other studies that confirmed such association [8, 13, 37].

In this study, the female sex was significantly negatively associated with a high risk for burnout among medical students in preclinical study years. Prior research is inconsistent—some studies did not find an association of sex with burnout syndrome [12, 20, 30, 34, 38–40], while other studies recorded a significantly higher level of cynicism in fourth-year male students compared to female students [41]. Maslach does not consider gender as one of the main burnout factors in employed persons, and considers that there are no differences between men and women or the differences are very small [42]. Some studies [12, 25] found no significant association of burnout with sex. On the other hand, research in Lebanon [8], India [43], Pakistan [38] confirmed a significant association between female sex and high level of burnout syndrome in medical students. Contrary to that, studies in Great Britain [34] and Saudi Arabia [44] have reported the opposite, suggesting that male students had a higher risk of burnout compared to female students. These results could be associated with the stereotypes of gender roles, but they could also be due to the presence of confounding/secondary associations between sex and certain other characteristics [45]. It is not clear why female students had a lower risk for burnout in this study. One of the possible explanations could be that the majority of students were female (nearly 70%), which could have somewhat attenuated the pressures that females have been facing in the last decades while trying to be equal with their colleagues, in order to prove themselves in fields controlled by males. Some studies have pointed out that, compared to males, females perceive challenging or threatening life events as stressful less often [46, 47], while other studies noted the opposite [48]. Also, studies suggest that, compared to male students, female students often have better social support and show rational choices in terms of priorities in life [46, 49]. Although one other study noted that female medical students at one Serbian University assessed their physical health status and general stress level as worse compared to males, differences between the sexes in the frequency of burnout syndrome were not found [23], which can be linked to more frequent doctor visits for advice and help and better health control in female medical students. The higher prevalence of high risk for burnout syndrome in male medical students than in female medical students in preclinical training may also be related to some other characteristics in male students that were not examined in this study, such as lack of financial resources, employment in different temporary jobs during the studies, providing support and care for their partners and family in a society where the traditional division into male and female roles still exists as well as many social roles inside and outside the faculty, which could be the reason for greater stress and higher risk of burnout syndrome in male medical students. More studies researching genetic and hormonal traits and what is their influence on the effect that gender has on the risk for burnout could help in understanding this association.

In this research, the association of smoking cigarettes with a high risk of burnout was noted in preclinical students of medicine. Until now, there have been relatively few studies that have investigated the association of smoking with burnout in students of medicine [34]. A few studies did not find a statistically significant association between burnout levels and smoking [20, 50]. Cecil [34] assessed medical students in Great Britain, and found no association of smoking and burnout, with smoking only significantly associated with high emotional exhaustion scores. A study in China, that surveyed 224 students of medicine, noted that burnout was associated with smoking [51]. These results indicate the existence of a potential relationship between smoking and burnout. But, since in most of the studies the number of those who were current smokers and ex-smokers was low in comparison with non-smokers, particularly in areas with successfully implemented national-level strategies for the control of smoking, further research is necessary to fully explain these results.

In this study, sedative use was independently associated with a high risk for burnout in students of medicine in clinical years. In Kragujevac, 26 (3.4%) students of medicine reported sedatives use. In a multicenter study in France [52], it was noted that medical students in the first year of studies used 1.5 times more anxiolytics compared to students of second-year: this was explained as a consequence of the pressure that students of first-year have to pass their first exams, which leads to increased levels of mood disorders and anxiety. As opposed to that, second-year students more often looked for sedative and stimulative effects, indicating greater troubles in terms of combining studies and social life. The change from preclinical to clinical training can be associated with worries related to wrong treatment, getting a disease from patients, dealing unintentional harm to patients, losing control over hours of sleep, performing clinical skills. It is not clear if the sedative use leads to more burnout or whether those students who already do not perform well and feel a high level of stress decide for recreative use of medications and drugs as a source of commodity [14]. Since education regarding the misuse of drugs, alcohol and illicit substances, constitutes a part of the basic curriculum of faculties of medicine [33, 53, 54], this association should be investigated in future studies.

Although this study showed that a high risk of burnout syndrome is present among medical students in Serbia, there was no increase in the prevalence of burnout with the transition from preclinical to clinical training. Despite the understanding that burnout syndrome is primarily related to academic stress, this study showed a strong association with behavioral factors (such as cigarette smoking and sedative use, and, to some extent, frequency of alcohol consumption). Our findings indicated that personal factors (male sex and behavioral factors) are linked to a high risk of burnout syndrome among medical students. The use of sedatives and cigarette smoking, as well as the frequency of alcohol consumption among medical students, raise concerns. Efforts to decrease burnout syndrome in medical students must address all of these elements, including the provision of adequate counseling to students as well as improvements in the teaching curriculum in the context of comprehensive knowledge of all the effects of exposure to the identified risk factors. The current literature regarding effective interventions to mitigate the impact of burnout syndrome in medical students is sparse [55, 56]. One study on burnout in medical students at different stages of the curriculum in Germany identified functional behavior-based coping strategies (including ’seeking support from friends’, ’seeking support from family’, ’doing relaxing exercise’, ’doing sports’ and ’seeking support from fellow students’), suggesting that social support and active exercises seem to be very important possibilities to reduce stress, exhaustion and burnout [57]. In a study looking for ways to prevent burnout among medical students at a university in Brazil, the prevalence of burnout syndrome was reduced in a group of students who frequently participated in a Balint prevention group, that is, in a group of participants who met regularly to present and discuss on cases to explore uncertainties and difficulties in the doctor-patient relationship [58]. However, additional research is needed to explore the possible causality of relationships described in this study, in order to determine the best approaches to prevent burnout syndrome in medical students.

### Strengths and limitations of the study

Based on the literature, this study is one of the first dedicated to identifying predictors of high risk of burnout in students of medicine in Serbia, and the only research that used the MBI-Student Survey to estimate burnout, which is a questionnaire validated for the population of Serbian medical students. Also, the response rate was high (90.9%). Still, our research had several limitations. Apart from the well-known shortcomings of cross-sectional design, a limitation is the use of self-reported data: even though the principle of anonymity was used during the survey, one cannot completely exclude information bias, because even though the privacy of all information was guaranteed, there is always the possibility that some respondents did not want to revel the symptoms of burnout. Further on, the study did not estimate the level of risk for burnout syndrome prior to enrollment to medical studies, which could have helped additionally in elucidating the effect of studies on burnout. Additionally, this research focused on personal characteristics, and not on variables related to the environment (e.g. interpersonal relations with colleagues and professors, etc.) and to curriculum, family history and other factors that could have influenced the occurrence of burnout syndrome in students of medicine and did not give data regarding other potential predictors of burnout syndrome (e.g. socio-economic status, etc.). Finally, even though the response rate was high, the issue of response bias could still exist, since persons who have burnout syndrome might not have attended the classes during the collection of data or might have decided against participating.

## Conclusions

In this study, the prevalence of high risk of burnout syndrome in medical students in preclinical and clinical grade was almost equal (in both groups almost equally about 15%). High risk for burnout syndrome in preclinical grade was correlated with male sex and cigarette smoking, while the use of sedatives was associated with burnout syndrome in medical students in clinical training. Assessing the prevalence of high risk for burnout and identifying factors associated with it in Serbian students of medicine expands the evidence base and provides a larger understanding of similar research. The correlation of burnout syndrome with some modifiable risk factors (such as cigarette smoking, use of sedatives, frequency of alcohol consumption) indicated the necessity of psychological support and counseling for medical students who are at high risk for burnout syndrome, which can have a large impact to their health, quality of life and professional performance. In addition, considering that teaching about the use and abuse of substances is part of the regular education of medical students, this association should be investigated in epidemiological longitudinal studies.
